# Pain Rehabilitation’s Effect on People in Chronic Pain: A Prospective Cohort Study

**DOI:** 10.3390/ijerph181910306

**Published:** 2021-09-30

**Authors:** Hafdís Skúladóttir, Amalia Björnsdottir, Janean E. Holden, Thóra Jenný Gunnarsdóttir, Sigridur Halldorsdottir, Herdis Sveinsdottir

**Affiliations:** 1 School of Health Science, University of Akureyri, Solborg v/Nordurslod, 600 Akureyri, Iceland; sigridur@unak.is; 2 School of Education, Faculty of Education and Pedagogy, University of Iceland, Stakkahlid 1, 105 Reykjavík, Iceland; amaliabj@hi.is; 3 School of Nursing, University of Michigan, Ann Arbor, MI 48109, USA; holdenje@umich.edu; 4 School of Health Science, University of Iceland, Eiríksgata 34, 101 Reykjavík, Iceland; thoraj@hi.is (T.J.G.); herdis@hi.is (H.S.)

**Keywords:** chronic pain, rehabilitation, sleep, self-management, health

## Abstract

Multidisciplinary long-term pain rehabilitation programs with a team of healthcare professionals are an integrated approach to treat patients with chronic non-malignant pain. In this longitudinal prospective cohort study, we investigated the long-term effects of multidisciplinary pain rehabilitation on the self-reported causes of pain, pain self-management strategies, sleep, pain severity, and pain’s interference with life, pre- and post-treatment. Eighty-one patients, aged 20–69 years, with chronic pain responded. The two most frequently reported perceived causes of pain were fibromyalgia and accidents. The difference in average self-reported pain severity decreased significantly at one-year follow-up (*p* < 0.001), as did pain’s interference with general activities, mood, walking ability, sleep, and enjoyment of life. At one-year follow-up, participants (21%) rated their health as good/very good and were more likely to state that it was better than a year before (20%). No change was found in the use of pain self-management strategies such as physical training at one-year follow-up. The intervention was effective for the participants, as reflected in the decreased pain severity and pain interference with life.

## 1. Introduction

The World Health Organization (WHO) has characterized chronic pain as the second-largest contributor to disability worldwide, with lower back pain being the single leading cause of disability [1]. Chronic pain is defined as an unpleasant sensory and emotional experience associated with, or resembling that associated with, actual or potential tissue damage and typically lasts longer than three months. Primary chronic pain refers to pain that is the presenting problem, such as with fibromyalgia or lower back pain. Secondary chronic pain is due to an identifiable cause, as in the case of chronic post-surgical or post-traumatic pain [2]. Chronic pain is often considered to be nociplastic pain or pain that arises from altered nociception despite no clear evidence of actual or threatened tissue damage [3]. This type of pain can occur in isolation (such as in fibromyalgia) or as part of a mixed-pain state (as in chronic lower back pain). The symptoms observed in nociplastic pain include widespread or intense pain (or both), fatigue, sleep, and mood problems [4]. In Iceland, the prevalence of chronic pain is as high as 48%, and among those with chronic pain, approximately 30% experience constant pain. The findings of this survey showed that the most common causes of chronic pain were myalgia, old trauma, rheumatism (e.g., rheumatoid arthritis and osteoarthritis), fibromyalgia, and migraines. Of those who reported chronic pain, 53.2% had consulted a healthcare provider for their pain, and rheumatism (as a perceived cause of pain) predicted pain-related healthcare utilization among women [5].

In Iceland, 27% of adults are obese, meaning that they have body mass indexes (BMIs) of 30.0 or higher [6]. Research has shown that being overweight or obese increases the likelihood of lower back pain, tension or migraine headaches, fibromyalgia, abdominal pain, and chronic widespread pain [7]. In turn, these conditions can affect the outcomes of pain rehabilitation programs [8]. Pain self-management strategies (e.g., medication, distraction, relaxation, activity pacing, and exercise) include specific tasks, activities, or methods that a person in chronic pain may employ in an effort to manage their symptoms and achieve certain goals, such as reduced pain interference with activities, mood, and relationships [9].

The present study focused on the effects of the three multidisciplinary pain rehabilitation programs in Iceland among participants with various causes of chronic pain. Multidisciplinary long-term pain rehabilitation programs (also called interdisciplinary pain rehabilitation) involve a team of healthcare professionals and an integrated approach to treat patients with non-malignant pain. These programs combine psychological interventions and physical training in cases where other interventions, such as pharmacological treatment or physiotherapy, are insufficient. While multidisciplinary rehabilitation programs do not always provide complete pain relief [10,11,12,13], they have been shown to improve life satisfaction and reduce pain severity as well as the negative psychological, social, and behavioral effects of pain [14,15]. For example, sleep difficulties are common among chronic pain patients. When pain and sleep are comorbid, both must be addressed to attain the maximum response to pain rehabilitation programs [16]. A recent systematic review and meta-analysis revealed that multidisciplinary rehabilitation lessens pain intensity and disability compared to active physical interventions, and these effects appear to be sustained in the long term [17].

Most studies on the effectiveness of multidisciplinary rehabilitation have involved patients with chronic lower back pain [17]. However, programs also exist for patients with various causes of pain [18], complex chronic non-malignant pain [13], or long-term symptoms following whiplash [19]. Based on the results of systematic reviews and meta-analyses of the best evidence regarding rehabilitation for chronic lower back pain patients, Malfliet et al. [20] recommended multidisciplinary rehabilitation programs and exercises that align with patients’ preferences and abilities. Furthermore, they found that exercise interventions have better, longer-lasting effects when combined with psychological components. The three multidisciplinary pain rehabilitation programs in Iceland provide both physical exercises combined with psychological components.

Four prior studies (each of which focused on a specific treatment at a single rehabilitation center) have examined the three aforementioned multidisciplinary programs, but none of these studies included all three programs within a single study, with the aim of examining their effects on pain severity and pain’s interference with the participants’ lives, health, and self-management strategies. One of these studies focused specifically on cognitive behavioral therapy for depression and anxiety in patients with chronic musculoskeletal pain. The results indicated that this intervention may enhance the long-term (up to three years) benefits of treatment, even though the participants reported little change in their pain intensity [21]. Another study focused on chronic pain patients’ participation in health assessment practices in a nursing context. Both chronic pain patients and their nurses participated in this study, and the results showed that using the assessment tool Hermes facilitated person-centered participation in the patients’ health assessments [22]. The third study used a combination of complementary therapies. The importance of the environment, the healing effects of nature, and opportunities for relaxation and distraction from normal life and daily stressors were highlighted in the findings. Furthermore, it was particularly important that the patients’ healing was self-motivated and self-directed [23]. The fourth and final study compared two interventions—a traditional multidisciplinary pain management program and neuroscience education with mindfulness-based cognitive therapy—for women with chronic pain. Pain intensity was measured with a visual analogue scale, and health-related quality of life was measured with the Icelandic Quality of Life scale. Both programs improved pain and health-related quality of life, but pain intensity lessened to a greater degree in the traditional program [24]. Further study is needed on the long-term effect of pain rehabilitation programs for chronic pain and the examination of variables that affect patient outcomes, such as self-managing strategies, sleep, evaluation of health, and sociodemographic variables.

The aim of the present study is to describe patients’ self-reported experiences of pain and investigate the long-term effects of multidisciplinary pain rehabilitation in Iceland. Specifically, this study aimed a) to explore and describe how individuals with chronic pain self-report their pain severity and pain’s interference with life before attending a multidisciplinary pain rehabilitation intervention (pre-treatment), on completion of the intervention (post-treatment), and one-year follow-up, and b) to explore changes in the participants’ pain self-management strategies, sleep, and health at one-year follow-up. Data were also gathered regarding perceived causes of pain, duration, and location.

## 2. Materials and Methods

### 2.1. Study Design and Setting

This longitudinal prospective cohort study aimed to investigate pain severity and pain’s interference with life in a sample of people with chronic pain attending a multidisciplinary pain rehabilitation intervention. The study settings included three rehabilitation centers in Iceland (Centers 1, 2, and 3) that provide multidisciplinary pain rehabilitation interventions. These centers are staffed with nurses, physicians, physiotherapists, psychologists, occupational therapists, social workers, nutritional consultants, massage therapists, and physical activity instructors. 

### 2.2. Participants

Participants were men and women in one of three Iceland pain rehabilitation centers. The emphasis of the study was on the intervention that the participants were to receive. Based on recommendations from the centers’ nurse unit managers and chief physicians, patients who did not attend the entire program, those who participated in a distance program, and those who had cancer were excluded from the study. The inclusion criteria for participation were chronic musculoskeletal pain lasting for at least three months; the ability to speak, understand, and read Icelandic; an age of 18–70 years (the investigated treatments are not offered to people older than 70 years of age); and admission to one of the three investigated rehabilitation centers.

Several reasons for exclusion were reported, such as a cancer diagnosis, program postponement, removal from the waiting list, not completing the program, and transferring to a distance program or another type of program. Those who withdrew from the study but met the inclusion criteria reported reasons such as not wanting to participate, not feeling up to it, inability to complete online questionnaires, sickness, and uncertainty as to whether they would attend the program. Final inclusion in the study comprised participants who completed the questionnaires (*n* = 81). A nearly equal number of participants attended the intervention at Center 1 (*n* = 39) and Center 2 (*n* = 38), but only four participants attended Center 3.

Permission to conduct the study was granted by the Icelandic National Bioethics Committee (VSN-15-101) and the chief physicians at the three investigated rehabilitation centers. The introductory letter given to the participants included information on the responsible parties and contact persons should they have any questions, comments, or concerns. The methodology was explained, and the participants were informed of their right to withdraw from the study whenever they chose.

### 2.3. Intervention

The intervention in the present study was a multidisciplinary pain rehabilitation program offered at the three investigated rehabilitation centers. The concepts of interventions, treatments, and programs are used interchangeably herein. The standard intervention was similar in all three centers, and treatment lengths ranged from four (Centers 2 and 3) to seven weeks (Center 1). The intervention begins and ends with assessing each patient’s condition. At the initial assessment, every patient is assessed to set goals and make decisions for the development of further rehabilitation procedures. The standard intervention includes scheduled individualized and group sessions with physical therapy (1–5 times a week), cognitive behavioral therapy (once a week), relaxation (3–7 times a week), aquatic exercise training (3–5 times a week), support, and education (5 times a week). A special focus is placed on self-management strategies and minimizing or reducing the use of pain medication. Lifestyle changes (e.g., more regular physical training, relaxation techniques, and learning how to better cope with pain) are also encouraged. The emphasis of the intervention is on education regarding different subjects related to pain and pain management, such as healthy lifestyle choices, goal setting, relaxation, stress management, sleep, medication, physical training, self-image, and coping. Two of the investigated centers (1 and 3) also offered mindfulness (3 times a week), massage (2 times a week), acupuncture (1–2 times a week), body awareness (1–2 times a week), and compassion-focused therapy (1 time a week) (Table 1). How often each week a session was applied depended on the evaluation of everyone’s needs. 

As described above, the three investigated centers offer similar (albeit not identical) multidisciplinary interventions. Due to the present study’s emphasis on the standard intervention, the small study population, and various causes of chronic pain, it was decided that the participants would be addressed as one cohort.

### 2.4. Procedure

Patients (*N* = 380) were screened by one contact person at each center (either the chief physician or a nurse unit manager) as soon as they were added to the waiting list for the program. Incoming patients (*n* = 236) then received a phone call from a research assistant, who introduced the study and provided instructions on how to participate. Those who agreed to participate received an introductory letter by mail, which contained a link and a password that enabled them to access and complete a questionnaire online. Those who responded to the first questionnaire (*n* = 144) received a second and third questionnaire (also online) if they fulfilled the inclusion criteria. A reminder was sent by email to those who did not respond within two weeks, a second reminder was sent one week later if there was still no response, and a final reminder was sent four weeks later. During the data collection process, 31 patients withdrew from further participation and 32 were excluded. Data were collected between September 2015 and February 2019.

### 2.5. Measures

The study questionnaires were based on those used previously [5] as well as questions developed specifically for this study. The questionnaires measured sociodemographic information, pain, pain characteristics, self-management strategies, sleep, and health.

#### 2.5.1. Sociodemographic Information

Demographic information was collected pre-treatment and included age (years), gender (male or female), education (compulsory, upper secondary, or higher), employment status (full-time, part-time, or other), marital status (married or living with a partner, single, divorced, or widowed), and BMI (kg/m^2^). Employment status and BMI were also measured at one-year follow-up.

#### 2.5.2. Perceived Causes of Pain

The participants were asked to indicate what they perceived to be the primary cause of their pain and whether they had been diagnosed or had some explanation for the causes of their pain (yes/no). Those who responded “yes” were asked to mark the causes of their pain on a list of possible causes of pain (e.g., fibromyalgia, myalgia, and disc prolapse).

#### 2.5.3. Pain Duration and Location

The participants were asked to report how long they had been in pain (years/months). They were also asked to indicate all areas of the body where they sensed pain by marking them on a list of 22 predefined anatomical areas of the body: (1) head, (2) face, (3) neck, (4) scapular/yoke upper back, (5) shoulder(s), (6) arm(s), (7) hand(s), (8) wrist(s), (9) finger(s), (10) upper back, (11) mid-back, (12) lower back, (13) chest, (14) hip, (15) hip joint, (16) groin, (17) abdomen, (18) pelvis, (19) foot/feet, (20) toe(s), (21) leg(s), and (22) knee(s).

#### 2.5.4. Pain Severity and Pain’s Interference with Life 

Pain severity and pain’s interference with life were measured with the Icelandic version of the Brief Pain Inventory (BPI; [25,26]). In studies by S. Gunnarsdottir et al. [26] and Jonsdottir et al. [5], the internal consistency of this measure was found to be α = 0.89 for the severity scale and α = 0.91 for the interference scale. The BPI includes three questions regarding pain severity during the previous 24 h, worst pain, least pain, and average pain. The fourth severity item measures current pain. Pain interference was evaluated by asking questions regarding the impact of any type of pain on seven aspects of daily life (e.g., “Mark one number that describes how, during the past 24 h, pain has interfered with your general activities, mood, walking ability, work, relations with other people, sleep, and enjoyment of life”). The participants rated their pain severity and pain interference on a 11-point scale (0 = “no pain” or “does not interfere” and 10 = “the worst pain imaginable” or “completely interferes”). According to Cleeland and Ryan [25], more daily activities are impaired as pain severity increases. For example, sleep, activity, mood, work, and life enjoyment are impaired when pain severity reaches Level 5. When pain severity reaches Level 7, the ability to walk is added to the list of impaired activities. Negative effects on relationships with others occur when pain severity reaches Level 8 [25].

#### 2.5.5. Self-Management Strategies

Participants were asked to indicate the measures that they took to relieve their pain (e.g., pain medications, NSAIDs, sedatives, regular physical training, heat/cold, relaxation, distraction, avoiding certain food/beverages, or positive thinking). They also indicated how often they used these measures on a 5-point scale (never, 1–3 times per month, 1–3 times per week, 4–6 times per week, or daily). A pain self-management strategy was used regularly and as recommended [27] if the participants reported using it four times or more (4–6 times per week or daily) for both time periods.

#### 2.5.6. Sleep

Quality of sleep was measured with three questions derived from the Pittsburgh Sleep Quality Index, a valid and reliable questionnaire [28]. The participants were asked to indicate for how many hours they normally slept per day. They were also asked to rate their quality of sleep over the past four weeks. The response options were (1) “I had no sleep problems at all,” (2) “I had some sleep problems,” (3) “I had many sleep problems,” and (4) “I had severe sleep problems.” Those who had experienced sleep problems in the previous month were asked to report whether they had experienced sleep problems due to pain.

#### 2.5.7. Health 

Two questions from the Short Form 36 Health Survey (SF-36v2) were used in this study. The participants evaluated their general health (excellent, very good, good, fair, or poor) and compared their current health to their health one year prior (much better now, somewhat better now, about the same, somewhat worse now, or much worse now; [29,30]). SF-36v2 has been widely used and the reliability and validity tested. For example, the reliability and validity of the instrument was tested and confirmed in another study in Iceland, where the internal consistency was acceptable, with Chronbach’s alpha of 0.78 for general health [31].

### 2.6. Statistical Analysis

The statistical analyses were conducted using the SPSS 27.0 statistical program (IBM SPSS Statistics for Windows, version 27.0. Armonk, NY: IBM Corp) [32]. Missing data were deleted according to a pairwise deletion procedure. Descriptive statistics (means, standard deviations, and percentages) were used to present the sample’s demographic, pain self-management, sleep, and health data. A Wilcoxon signed-rank test was used to compare the participants’ pre-treatment self-evaluation of their health with their evaluations at one-year follow-up. A related-samples McNemar change test was used to detect differences in sleep problems due to pain and the use of various pain self-management strategies (four times a week or more) between pre-treatment and one-year follow-up (Table 5). A paired *t*-test with bootstrapping was used to detect differences in pain severity and pain interference between pre-treatment and one-year follow-up. Differences in pain severity and pain interference were interpreted using Cohen’s *d* as small (0 to 0.2), medium (0.3 to 0.7), and large (>0.8) (Table 4). The level of significance established for this study was set at *p* < 0.05.

## 3. Results

### 3.1. Characteristics of the Sample (n = 81)

The respondents’ ages ranged from 20 to 68 years (*M* = 47.2 years, *SD* = 11.9 years). Most of the respondents were women (84%), 38% had completed upper secondary education, 27% had completed higher education, and 38% were working (24% full-time and 14% part-time). Most of the participants were married or living with a partner (77%). At one-year follow-up, 34% of the participants were working (20% full-time and 14% part-time). The “other” employment status included participants who were unemployed, disabled, students, homemakers, or self-employed. The average BMI was 30.6 (*SD* = 7.2) pre-treatment and 30.8 (*SD* = 6.6) at one-year follow-up. The participants’ sociodemographic characteristics are listed in Table 2.

### 3.2. Perceived Pain Causes, Duration, and Locations

The participants presented with diverse causes of pain, duration, and location. As shown in Table 3, the most frequently reported perceived cause of pain was fibromyalgia (*n* = 40), followed by accidents (*n* = 36), myalgia (*n* = 33), and disc prolapse (*n* = 24); however, most of the participants reported more than one cause of pain. Pre-treatment, most of the participants (*n* = 72, 89%) reported that they had received an explanation or diagnosis for their pain.

The mean pain duration was 10.3 years (range: 1–55 years). The most frequently reported location of pain was the lower back (*n* = 63, 80%), followed by the shoulder(s) (*n* = 56, 71%). Most of the participants (*n* = 76, 94%) reported pain in more than one location (Table 3).

### 3.3. Pain Severity and Pain’s Interference with Life

The participants rated their pain severity significantly lower at post-treatment and at one-year follow-up compared to pre-treatment (Table 4). Average self-reported pain severity decreased significantly from pre-treatment to one-year follow-up (*p* < 0.001) (medium effect), giving an estimate of the long-term effect of the treatment. In addition, there was a significant reduction in self-reported estimates of the worst pain (*p* = 0.041) and current pain (*p* = 0.048) from pre-treatment to one-year follow-up (small effect) (Table 4).

Average self-reported pain interference decreased from pre-treatment to post-treatment and decreased significantly for most items (all except for the ability to work and relations with other people) from pre-treatment to one-year follow-up. The average differences in pain interference between pre-treatment and one-year follow-up were statistically significant for general activities (*p* = 0.007), mood (*p* = 0.012), walking ability (*p* = 0.034), sleep (*p* = 0.035), and enjoyment of life (*p* = 0.004) (small to medium effect). The observed differences in self-reported pain severity and pain’s interference with life are listed in Table 4.

### 3.4. Pain Self-Management Strategies

The three most common pain self-management strategies used by participants four or more times per week pre-treatment were positive thinking (68%), medication (58%), and distraction (58%). No significant difference in the proportion (or percentage) of participants who used these strategies was found between pre-treatment and one-year follow-up (Table 5).

### 3.5. Sleep

As shown in Table 5, 90% of the participants reported sleep problems due to pain pre-treatment vs. 83% at one-year follow-up; however, this reduction was not statistically significant (*p* = 0.146). Furthermore, the average total hours of sleep did not change between pre-treatment (*M* = 6.9 h, *SD* = 1.6 h) and one-year follow-up (*M* = 7.0 h, *SD* = 1.5 h).

### 3.6. Health

Importantly, at one-year follow-up, 21% of the participants reported that their health was good/very good (vs. 7% pre-treatment). Furthermore, 20% stated that their health was much better at one-year follow-up than one year prior (vs. 4% pre-treatment: Table 5)

## 4. Discussion

The aim of the current study was to describe patients’ self-reported experiences of pain and investigate the long-term effects of a multidisciplinary pain rehabilitation intervention offered by three main programs in Iceland.

One of the most significant findings in the current study was that the intervention appeared to influence the participants’ self-reported pain in a positive manner. The participants’ self-reported pain was significantly lower at one-year follow-up than pre-treatment. These results were similar to those of other studies, which have shown that multidisciplinary rehabilitation programs reduce pain intensity [15,33]. However, pain intensity was still high (around 6–8) and the least pain had decreased post-treatment, but at one-year follow-up, it was the same as pre-treatment (around 4.5).

Pain’s interference with walking ability and general activities differed significantly between pre-treatment and one-year follow-up in the current study. Surprisingly, the participants had not used any particular pain self-management strategy more frequently than any other at one-year follow-up. Even regular physical training (an emphasis of the intervention) was used as a method of pain self-management with the same frequency at one-year follow-up as pre-treatment. This result was similar to the findings of a study by Dysvik et al. [34], in which training activities were similar at the starting point and at 12-month follow-up. This could be explained by the fact that the participants had less time to train regularly in their daily routine at home than they had while participating in the program, in which they could focus entirely on themselves and their needs and take a break from their normal lives and daily stressors [23]. Physical training is an important method of pain self-management and it is possible that an extended period in the intervention or more follow-up is needed. This needs to be studied further.

Participating in the intervention positively influenced self-reported health. More participants rated their health as good or very good at one-year follow-up than they did pre-treatment, and many stated that their health was much better than before the program; this finding was similar to the findings of other studies [13,14,35]. It is likely that positive thinking, the strategy used most by participants, influenced this positive view of their health. This result is similar to the findings of a study by Dysvik et al. [36], in which 81% of participants with chronic pain reported positive and important changes after multidisciplinary pain rehabilitation, in part due to positive thinking. The importance of positive thinking was also underscored by a study conducted by Wideman et al. [12], in which patients with chronic pain experienced high levels of negative pain-related factors (e.g., disability) while simultaneously taking steps toward personal growth; these participants looked at growth positively instead of concentrating on information that was not useful, as focusing on negative information only caused them frustration.

Mental disorders (e.g., depression, anxiety, and suicidal thoughts) are highly prevalent in chronic pain conditions, which often affect mood and subjective enjoyment of life [20,37]. In the current study, pain’s interference with mood and enjoyment of life was reduced one year after the intervention was completed. However, it was still high (around 6), which raises the question of whether it is time to reorganize the intervention and start to plan more follow-up with support, education, and assessment of mental conditions.

Sleep deprivation is a risk factor for chronic pain [16]. Pain’s interference with sleep was reduced in the current study. Total sleep time was the same at one-year follow-up as it was pre-treatment. While sleep problems due to pain were slightly lower at one-year follow-up than they were pre-treatment, this difference was not significant. The results of Davin et al. [16] showed that a stronger association between the previous night’s total sleep time and next-day pain contributed to the greatest overall treatment benefits in terms of pain reduction and total sleep time. This raises the question of whether enough is done in the intervention to deal with sleep problems in connection with pain. Further study is needed to explore the effect of the intervention regarding sleep and to specifically target sleep problems due to pain [16,20].

One unexpected finding in the present study was that pain’s interference with work decreased one year after the intervention was completed but not significantly. The proportion of participants who worked full-time or part-time did not change significantly from one-year follow-up (34%) to pre-treatment (38%). The proportion of working participants in this study was higher than in a study by Silvemark et al. [15], in which the proportion of participants who described their source of income as paid work was 27.3% at admission and 25% at one-year follow-up. The reasons for this difference are not clear, but it may be that the majority of participants were younger than 50 years of age (58%), with either upper secondary or higher education (65%), which might have given them more opportunities to find jobs. Additionally, pain’s interference with relations with others was also reduced at one-year follow-up, but not significantly in the current study. This difference may be explained by differences in marital status since most of the participants (77%) were either married or lived with a partner. 

Weight (especially increased BMI) has been studied in connection with chronic pain and chronic pain treatment [20]. It is known that comprehensive pain rehabilitation programs improve physical and psychological functioning in patients in as little as three weeks, regardless of weight status [38]. However, following outpatient physical therapy, disability improved in overweight patients but not in obese patients [39], and severely obese subjects showed less improvement than the non-obese subjects following an interdisciplinary treatment program aimed specifically at patients with fibromyalgia [8]. In the current study, most of the participants were obese both pre-treatment and at one-year follow-up, and no associations were found between BMI and any other variables. Because studies have shown reduced pain in chronic lower back pain patients after a nonsurgical weight loss program involving physical exercise and changes in dietary behavior [20], it may be time to place more emphasis on weight loss and physical exercise in pain rehabilitation.

One non-significant finding of the present study was that the participants used less medication for pain relief at one-year follow-up than they did pre-treatment. A follow-up study with greater statistical power than the present study should be undertaken to further examine this finding. This result tends to support the findings of Saltychev et al. [40], in which the purchase of prescription medication decreased significantly following a one-year rehabilitation program emphasizing analgesics, and the work of Norrefalk and Borg, in which the use of any analgesics decreased significantly after one year following an eight-week rehabilitation study [11]. However, specific questions regarding medication usage, type, and duration were not addressed in the current study and this area requires further study.

The best solution for people with chronic pain may be to reinforce the use of pain self-management strategies over a longer period by implementing better follow-up strategies after they complete pain rehabilitation. Other researchers have concluded that there is a need for an individualized form of follow-up with several intervention options [18,41,42]. For example, a program could be developed in which the patient can choose between in-person, technology-assisted [43], and Internet-based self-management activities to reduce their pain and improve their quality of life [44]. Furthermore, the ability to choose between regular contact through apps [45], telephone calls, chat rooms, support groups [41], and consultations at community health centers could also provide individualization.

## 5. Strengths and Limitations

The present study took place in a small country and lacked the power of large multisite studies. Nevertheless, large communities are composed of smaller communities, which often mirror the larger communities of which they are a part. Consequently, the description and analysis of small communities are relevant in the larger context.

One strength of this study was that it investigated a formal pain rehabilitation intervention in a single country. None of the members of the research group were part of the group of staff members at the investigated centers. The intervention was effective in several areas of pain management, which is a valuable finding. No control groups were used because it was expected that there would be clear differences between the effects of the interventions. The small number of participants from Center 3 has decreased the significance of some of the findings. The high proportion of dropouts in this study is also acknowledged. Although the reasons for withdrawal were not systematically addressed, several explanations were supplied by some of the non-responders.

Finally, the sample size of the present study was relatively small, resulting in the study’s relatively low statistical power. It is impossible to state whether the patients could maintain the changes that they achieved in this study for more than a year.

When participants were asked to indicate what they perceived to be the cause of their pain, the most commonly reported causes were fibromyalgia, accidents, myalgia, and disc prolapse. In the current study, 94% of the participants had pain in more than one location, which may have made it difficult to answer questions concerning pain severity. Therefore, the participants were not asked specifically about pain in each location but were asked about pain in general. Future studies could be developed that study the role of multidisciplinary rehabilitation programs on more specific types of pain conditions.

## 6. Implications for Pain Rehabilitation

The results of this study indicate that follow-up after the completion of pain rehabilitation could help patients to continue to engage in healthy lifestyle activities (e.g., regular physical training for pain relief) four or more times a week. This does not necessarily mean that all patients should return to their rehabilitation centers; rather, patients could choose between attending these centers and using some form of online or technical assistance several months after the intervention. The health professionals in the present study assessed the health status of each patient before the intervention, during the intervention, and at the intervention’s completion. It could be valuable to screen for and remain aware of chronic pain patients’ perceived causes of pain, BMIs, pain self-management strategies, levels of pain severity, and levels of pain interference with life. Furthermore, health professionals can provide education and support through rehabilitation centers, community health centers, apps, chat rooms, Zoom, videoconferencing, or telephone calls. The findings and implications of the present study must be studied further with more statistical power to determine the effects of the examined intervention over longer periods of time.

## 7. Conclusions

The multidisciplinary pain rehabilitation program of three major centers in Iceland was effective in decreasing pain severity and pain’s interference with general activities, mood, walking ability, sleep, and enjoyment of life in subjects with a wide range of chronic pain problems. Moreover, the participants experienced improved health post-intervention. However, the participants did not maintain regular physical training at one-year follow-up, and their sleep problems due to pain did not change over the course of the intervention. Follow-up is recommended after pain rehabilitation interventions, and the participating health professionals are in a strong position to provide education and support at community health centers, at rehabilitation centers, or through some form of online or technical assistance. These findings support the effectiveness of multidisciplinary rehabilitation programs for pain and will be used to guide further research.

## Figures and Tables

**Table 1:** Description of standard intervention.

Treatment Options	Center 1	Center 2	Center 3
**Standard intervention in three centers**			
Treatment length	5–7 weeks	4 weeks	4 weeks
Cognitive behavioral therapy for each individual	×1/week	×1/week	×1/week
Assessment	×1/week	×2/week	×1/week
Support and education	×5/week	×5/week	×5/week
Balance in daily life	×4/week	×5/week	×4/week
Relaxation in groups	×3/week	×4/week	×5–7/week
Physical therapy	×1–5/week	×1–5/week	×1–2/week
Group training with physiotherapist	×1–5/week	×5/week	×4/week
Group training with nurse or occupational therapist	×1–5/week	×2/week	×3/week
Aquatic exercise training	×5/week	×4/week	×3/week
**Other treatment options in two centers**			
Compassion focused therapy	×1/week		×1/week
Mindfulness	×1/week		×3/week
Massage	×1/week		×2/week
Acupuncture	×1/week		×1–2/week
Body awareness	×1/week		×2/week
**Other treatment options in one center**			
Cognitive behavioral therapy in groups	×1/week		
Health bath			×1/week
Knipp water therapy			×2–5/week
Mud			×2/week
Meditation			×1/week

**Table 2:** Description of sociodemographic variables (*n* = 81).

Variables	*n*	%
Gender	81	
Females	68	84
Males	13	16
Age	81	
40 years or less	24	30
41–50 years	23	28
51 years or older	34	42
Education	79	
Compulsory	28	35
Upper secondary	30	38
Higher	21	27
Employment status	80	
Full-time	19	24
Part-time	11	14
Other	50	62
Marital status	80	
Marriage/living with a partner	62	77
Single/divorced/widowed	12	15
BMI	77	
Underweight	4	5
Healthy weight	13	16
Overweight	19	25
Obese	41	54

**Table 3:** Causes of pain and pain location (*n* = 81).

	*n*	%
**Causes of pain**		
Fibromyalgia	40	49
Accidents	36	44
Myalgia	33	41
Disc prolapse	24	30
Osteoarthritis	20	25
Cartilage destruction	12	15
Whiplash	12	15
Migraine	12	15
Chronicfatigue syndrome	11	14
Violence	7	9
Rheumatoid arthritis	5	6
Polymyalgia rheumatica	2	2
Psoriasis arthritis	2	2
Osteoporosis	1	1
Rheumatoid spondylitis	1	1
**Location**		
Lower back	63	80
Shoulder(s)	56	71
Scapular	51	65
Neck	49	62
Hip	46	58
Foot/feet	44	56
Leg(s)	44	56
Knee(s)	38	48
Hand(s)	37	47
Upper back	36	46
Mid back	36	46
Head	36	46
Arm(s)	36	46
Finger(s)	36	46
Hip joint(s)	33	42
Toe(s)	25	32
Wrist(s)	24	30
Chest	21	27
Groin(s)	18	23
Abdomen	15	19
Face	15	19
Pelvis	13	16
**Number of locations**		
0–5	24	30
6–10	21	26
11–15	20	25
16–22	16	19

**Table 4:** The differences in self-reported pain severity and pain interference.

		Pre-Treatment	Post-Treatment	One-Year Follow-Up	Pre-Treatment/One-Year Follow-Up	Pre-Treatment/One-Year Follow-Up
	*n*	*M (SD)*	*M (SD)*	*M (SD)*	*p*-Value *	*Cohen d*
**Pain severity**						
Worst now	79	7.4 (1.78)	6.9 (2.07)	6.9 (2.08)	**0.048**	0.23
Worst	79	8.4 (1.56)	7.6 (1.97)	7.9 (1.97)	**0.041**	0.23
Least	79	4.5 (1.93)	4.1 (2.02)	4.4 (2.01)	0.517	0.07
Average	79	6.6 (1.65)	5.9 (1.90)	5.9 (1.83)	**0.001**	0.42
**Pain interference**						
General activity	76	7.7 (2.11)	6.5 (2.49)	6.7 (2.63)	**0.007**	0.32
Mood	79	6.7 (2.71)	5.3 (2.46)	5.8 (2.52)	**0.012**	0.29
Walking ability	78	6.6 (3.02)	5.6 (2.75)	5.9 (2.89)	**0.034**	0.24
Work	75	8.4 (2.90)	7.9 (3.13)	7.9 (3.24)	0.190	0.15
Relations with other people	78	6.0 (3.03)	4.9 (2.67)	5.3 (2.78)	0.079	0.21
Sleep	77	7.6 (2.86)	6.2 (2.84)	7.0 (2.80)	**0.035**	0.24
Enjoyment of life	79	7.6 (2.28)	5.7 (2.71)	6.5 (2.67)	**0.004**	0.34

* Values in bold indicate statistically significant differences (*p* < 0.05). *M* = mean; *SD* = standard deviation. Paired *t*-test bootstrap was only used for differences between pre-treatment and one-year follow-up.

**Table 5:** The differences in self-managing strategies, sleep, and health (*n* = 81).

	Pre-Treatment *n*	%	One-Year Follow-Up *n*	%	*p* Value *
**Self-managing pain** **4 times or more per week**					
Positive thinking	48/71	68	55/71	77	0.167
Medication	43/74	58	37/74	50	0.307
Distraction	40/69	58	41/69	59	1.00
Regular physical training	25/74	34	26/74	35	1.00
Avoid certain foods/beverages	23/73	30	31/71	44	0.181
Relaxation	20/66	30	21/66	32	1.00
Heat/cold	18/69	26	20/69	29	0.804
**Sleep**					
Sleep problems due to pain	73/81	90	67/81	83	0.146
**Health**					
Very good	0/81	0	3/79	4	
Good	6/81	7	13/79	17	
Fair	28/81	35	47/79	60	
Poor	47/81	58	16/79	20	**<0.001**
**Comparing health to one year ago**					
Much better now	3/81	4	16/79	20	
Somewhat better now	14/81	17	21/79	27	
About the same	22/81	27	24/79	30	
Somewhat worse now	25/81	31	14/79	18	
Much worse now	17/81	21	4/79	5	**<0.001**

* Values in bold indicate statistically significant differences (*p* < 0.05). Related-samples McNemar change test was used to compare the difference in sleep problems due to pain and in using various pain self-management strategies 4 times per week or more between the two time points. Wilcoxon signed-rank test was used to compare evaluation of health and compare health to one year ago at pre-treatment and one-year follow-up.
